# Supplementary material for: Intracellular Delivery of Peptides and Proteins with an Engineered Membrane Translocation Domain
Source: ACS Chem Biol. 2026 Jul 1;21(7):1818–28. doi: 10.1021/acschembio.6c00383 (PMC13390933; doi:10.1021/acschembio.6c00383)
Supplement: Supplementary file 1 [file cb6c00383_si_001.pdf]

Supporting Information

Intracellular Delivery of Peptides and Proteins with an Engineered Membrane Translocation Domain

Prabhat Bhat[1], Heba Salim[1], Jeremy L. Ritchey[1], Na Li[2], Brendan Harty[1], Thomas Patel[1], Jing Zhao[3,4], Qi-En Wang[2,5], Virginia L. King[6], Louis Tartaglia[6], Jenő Gyuris[6], and Dehua Pei[1]*

*E-mail: pei.3@osu.edu

Table of Contents

Experimental Details……………………………………………………………………S2
References………………………………………………………………………………S8
Figures S1-9……………………………………………………………………………S9
Table S1………………………………………………………………………………..S20

Experimental Details

Molecular Cloning and Mutagenesis. The DNA sequence encoding the 10th human fibronectin type III (FN3) domain was synthesized (GenScript) and cloned into the prokaryotic expression vector pET-15b. The construct contained an N-terminal hexahistidine tag followed by a thrombin cleavage site to enable purification by immobilized metal affinity chromatography (IMAC). A flexible $(GGS)_3$ linker and a unique cysteine were added at the C terminus to permit site-specific labeling with thiol-reactive agents. A *Sac*I restriction site was inserted between the linker and cysteine to allow fusion of cargo proteins at the C terminus. Similarly, the LgBit–mCherry gene fragment was synthesized (Twist Bioscience) and cloned into the pET-15b vector.

MTD1–5 variants were generated from plasmid pET-15b-FN3 by QuikChange site-directed mutagenesis.[57] The R111T mutant (MTD4s) was synthesized (Twist Bioscience) and cloned into the pET-15b-FN3 backbone, while the thrombin cleavage site was subsequently removed by QuikChange mutagenesis. HiBiT-tagged constructs (FN3-HiBiT, MTD2-HiBiT, and MTD4-HiBiT) were created by inserting the 11-amino acid HiBiT tag immediately after the $(GGS)_3$ linker and removing the C-terminal cysteine by PCR.

C-Terminal fusion constructs were generated by inserting gene fragments downstream of MTD4, including those encoding PTP1B (residues 1–321), RBDV, and EGFP-NLS-Cre, using the *Sac*I and other compatible restriction sites. For MTD4-PTP1B, the $(GGS)_3$ linker was replaced with a rigid $(EAAAK)_2$ linker to minimize intramolecular interactions between MTD4 and PTP1B. The catalytically inactive variant, MTD4-PTP1B(C215S), was generated by QuikChange mutagenesis.

ASL-MTD4 and ASL constructs were synthesized and cloned by WuXi AppTec. MEC and EC constructs were generated by inserting two nuclear localization signals (NLS) at the C terminus of Cre. The identity of all DNA constructs was confirmed by sequencing of the complete coding region. All protein sequences and DNA primers used in this work are listed in Table S1.

Protein Expression and Purification. *E. coli* BL21(DE3) cells transformed with the appropriate plasmid were grown in LB medium supplemented with 75 μg/mL ampicillin at 37 °C. At an OD_{600} of 0.6–0.8, protein expression was induced with 0.5 mM isopropyl β-D-1-thiogalactopyranoside (IPTG) for 4 h at 37 °C. Cells were harvested by centrifugation. For FN3, the cell pellet was resuspended in 50 mL of lysis buffer [25 mM Tris, pH 7.4, 150 mM NaCl, 25 mM imidazole, 3 mM β-mercaptoethanol, protease inhibitor cocktail tablets (Roche), and 0.2 mg/mL lysozyme]. The lysate was sonicated briefly and centrifuged at 35,000g for 20 min at 4 °C. The clarified lysate was loaded onto a Ni-NTA column attached to an ÄKTA FPLC system (Cytiva). The column was washed extensively with wash buffer (25 mM Tris, pH 7.4, 300 mM NaCl, 10% glycerol, and 50 mM imidazole). Bound protein was eluted with a linear gradient of 50–500 mM imidazole in wash buffer. MTD1-5, MTD4s, RBDV, MTD4-RBDV, MTD4-PTP1B, MTD4-PTP1B(C215S), PTP1B, and LgBit-mCherry were purified similarly. FN3-HiBit, MTD2-HiBit, and MTD4-HiBit were expressed in *E. coli* as inclusion bodies, as attempts to purify these fusion proteins from the soluble fraction resulted in rapid proteolysis of the unstructured HiBit motif. After lysis and centrifugation, the inclusion bodies were washed twice with wash buffer containing 1 M urea and 1% Triton X-100, solubilized in wash buffer containing 8 M urea (solubilization buffer), and loaded onto a pre-equilibrated Ni-NTA column. On-column refolding was achieved by applying a linear gradient from 8 M to 0 M urea over 2 h, followed by washing

with refolding buffer (wash buffer supplemented with 5 mM β-cyclodextrin). The refolded protein was eluted the next day with a linear gradient of imidazole as described above.

Proteins MEC and EC were expressed in *E. coli* BL21(DE3) Rosetta pLysS cells grown in LB medium supplemented with 75 μg/mL ampicillin and 35 μg/mL chloramphenicol at 37 °C until OD_{600} reached 0.6. Protein expression was induced with 0.5 mM IPTG at 18 °C for 20 h. The soluble lysate was applied to a pre-equilibrated SP FF (strong cation exchanger) column. The column was extensively washed with low-salt phosphate buffer (10 mM Na_2HPO_4, 1.8 mM KH_2PO_4, 50 mM NaCl, and 2.7 mM KCl at pH 6.5). Proteins were eluted with a linear gradient of 0.05–1.5 M NaCl in the phosphate buffer. ASL and ASL-MTD4 were purified at WuXi AppTec. Briefly, proteins were expressed in *E. coli* BL21(DE3) cells as described above. Cell pellets were lysed in 25 mM HEPES (pH 7.8), 600 mM NaCl, 1% Triton X-100, 10% glycerol, and 1 mM tris(carboxyethyl)phosphine (TCEP), supplemented with a protease inhibitor cocktail and benzonase. The clarified lysate was applied to a Ni-NTA affinity column pre-equilibrated with lysis buffer. The column was washed sequentially with HEPES buffer containing 600 mM NaCl, 1 mM TCEP, and 10% glycerol with stepwise increases in imidazole concentration (up to 50 mM). Bound proteins were eluted in 25 mM HEPES (pH 7.8), 150 mM NaCl, 150 mM arginine, 1 mM TCEP, 10% glycerol, and 500 mM imidazole. Eluted proteins were further purified by gel-filtration chromatography on a HiLoad 16/600 Superdex 200 pg column (Cytiva) equilibrated in 25 mM HEPES (pH 7.8), 150 mM NaCl, 150 mM arginine, 1 mM TCEP, and 10% glycerol.

All proteins were purified to near homogeneity, as assessed by SDS-PAGE. Proteins were concentrated and exchanged into sterile buffer (25 mM Tris-HCl or HEPES, pH 7.4, 150 mM arginine, 150 mM NaCl, 1 mM TCEP, and 30% glycerol). MEC, EC, ASL-MTD4, and ASL proteins were treated with Pierce high-capacity endotoxin removal resin (ThermoFisher) to remove endotoxins. Endotoxin levels were determined with the Pierce chromogenic endotoxin quantitation kit (ThermoFisher). Protein concentrations were determined by the Bradford assay as well as absorbance at 280 nm. Purified proteins were aliquoted, flash-frozen, and stored at −80 °C.

TMR Labeling. FN3 (or MTDs) was incubated in a buffer solution (25 mM Tris, pH 7.4, 150 mM NaCl, 5 mM TCEP, and 30% glycerol) containing 8 equiv of TMR-5-maleimide for 2 h at room temperature with constant mixing. Excess TMR dye was removed by passing the solution through a desalting column (Bio-Rad; 7-kDa molecular weight cut-off). The absorbances at 280 and 544 nm were measured on a UV-vis spectrophotometer and the degree of labelling (DOL) was calculated by using equation

$$\mathrm{DOL} = \frac{A_{544} * \varepsilon_{protein}}{\left(A_{280} - (A_{544} * \mathrm{CF})\right) * \varepsilon_{dye}}$$

where A_{544} and A_{280} are absorbances at 544 and 280 nm, respectively, and ε is the extinction coefficient and CF is the correction factor of the dye. The DOL values were 0.55, 0.67, and 0.52 for FN3, MTD2, and MTD4, respectively.

Peptide Synthesis. Peptides were synthesized on a Liberty Blue microwave-assisted peptide synthesizer and Wang resin by using 10 equiv of diisopropylcarbodiimide, 5 equiv of Oxyma pure, and 5 equiv of Fmoc-amino acids. Each coupling reaction was carried out at 90 °C for 4 min, except for arginine which was coupled twice at 90 °C for 4 min. N-Terminal acetylation was

performed by incubating the resin with 10 equiv of acetic anhydride and 10 equiv of diisopropylethylamine (DIPEA) in dichloromethane for 15 min at room temperature. For CPP12-HiBit, peptide cyclization was carried out as previously described.[23] Peptides were cleaved off the resin and deprotected by the treatment with a 91:3:3:3 (v/v) solution of trifluoroacetic acid, 2,2′-(ethylenedioxy)diethanethiol (DODT), H_2O, and triisopropylsilane at room temperature for 3 h. The crude peptide was precipitated in cold diethyl ether and purified by reversed-phase HPLC on a Waters C18 column. Purity (>95%) and authenticity of the peptides were confirmed by LC/MS on a Waters Acquity UPLC system equipped with an SQD2 electrospray ionization mass spectrometer.

Cell Culture. HeLa and HEK293T cells were cultured in Dulbecco's Modified Eagle Medium (DMEM), H358, H1915, and H1299 cells in Roswell Park Memorial Institute (RPMI) medium, and GM01661 and GM00525 cells in Eagle's Minimum Essential Medium (EMEM) with Earle's salts and non-essential amino acids with 2 mM L-glutamine. Each medium was supplemented with 10% fetal bovine serum (FBS) and 1% penicillin/streptomycin. Primary cells obtained from the lung tissues of Ai14 mice (JAX stock #007914) were grown in DMEM supplemented with 20% FBS, 1 mM sodium pyruvate (Lonza), 1x MEM non-essential amino acid solution (HyClone), and 1% penicillin/streptomycin. All cell cultures were maintained in their respective media in a humidified incubator at 37 °C in the presence of 5% CO_2, unless mentioned otherwise. Adherent cells were incubated with 0.25% trypsin-EDTA for 5 min at 37 °C in the presence of 5% CO_2 during subculturing and harvesting.

NanoLuc Complementation Assay. HEK293T cells (6.0 x 10^5 cells/well) were seeded in a 6-well plate. Next day, the cells were transfected with 0.5 μg of LgBit expression vector (Promega) using lipofectamine 2000 (ThermoFisher) for 24 h according to manufacturer's protocol. The cells were reseeded (10,000 cells/well) in a poly-D-lysine coated 96-well plate. The cells were treated with various concentrations of HiBit or peptide-/protein-HiBit conjugates in media supplemented with 1% FBS and 1% penicillin/streptomycin for 4 h. The cells were washed twice with DPBS and incubated in 100 μL of Opti-MEM and 25 μL of NanoLuc reagent per well (Promega, Cat#N2012). Luminescence was measured immediately on a Tecan Infinite M1000 Pro microplate reader and plotted against the peptide/protein concentration using GraphPad Prism 6.

Because peptide-/protein-HiBit conjugates may have different complementation efficiencies inside the cell, the complementation efficiencies of the conjugates were determined by carrying out the complementation assay in crude cell lysates. Thus, after re-seeding the cells in a 96-well plate, the cells were treated with 5 nM HiBit or a protein-/peptide-HiBit conjugate in Opti-mem supplemented with 0.01% (w/v) digitonin (Sigma-Aldrich). After 15 min, 25 μL of NanoLuc reagent was added into each well and luminescence was measured immediately on a Tecan Infinite M1000 pro microplate reader. The complementation efficiency of each peptide-/protein-HiBit conjugate (relative to that of HiBit, 100%) was calculated from the ratio of RLU of the conjugate over that of HiBit (Figure S1).

The cytosolic delivery efficiencies reported in Figure 2c represent the experimental RLU values from above, after correction for their relative complementation efficiencies.

Confocal Microscopy. HeLa cells were seeded in a 35/10-mm glass-bottom microwell dish with four compartments at a density of 5.0 x 10^4 cells/mL. The cells were washed twice with DPBS and treated for 2 h with vehicle control (buffer only) or 5 μM TMR-labeled protein and 5 μM AlexaFluor647-conjugated dextran in DMEM supplemented with 1% FBS and 1% penicillin/streptomycin. The cells were washed twice with DPBS, counterstained with Hoechst 33342 (Invitrogen, Cat#H3570), supplemented with phenol red-free DMEM containing 1% FBS and 1% penicillin/streptomycin, and imaged. MTD4-HiBiT or HiBiT was incubated with 1.5 equiv of LgBiT-mCherry for 30 min to allow NanoLuc complementation. HeLa cells, seeded as described above, were treated with the protein complexes or vehicle control for 4 h in serum-free DMEM supplemented with 5 μM AlexaFluor647-conjugated dextran. After treatment, cells were washed twice with DPBS and counterstained with LysoTracker™ Deep Red (Invitrogen, Cat#L12492) and Hoechst 33342 before imaging. Live-cell confocal microscopy was performed on a Nikon A1R confocal microscope (ECLIPSE Ti-E automated, inverted) equipped with 60x water-, 60x and 100x oil-immersion objectives. NIS Elements AR was used for image analysis. Manders' overlap coefficients and Pearson's correlation coefficients were calculated using the JaCoP plugin in ImageJ with identical analysis settings applied to all images.[58]

High Throughput Thermal Scanning (HTTS). FN3 and MTD4 proteins were prepared at concentrations of 0.25, 0.5, and 1 mg/mL. SYPRO® Orange dye (Invitrogen) was added to each sample to a final concentration of 15×. The mixtures were loaded into iCycler 96-well 0.2 mL thin-wall PCR plates and sealed with iCycler optical-quality sealing tape (Bio-Rad). Thermal denaturation was performed using the iCycler Melt Curve script (0.2 °C every 12 s), with fluorescence monitored using a 490 ± 10 nm excitation filter (from the SYBR Green set) and a 575 ± 10 nm emission filter (from the HEX filter set). Data were processed and plotted as previously described.[59]

Serum Stability Assay. Protein sample (final concentration 10 μM) was mixed with 25% clarified human serum (total volume 200 μL) and incubated at 37 °C. At varying time points (0–24 h), 20-μL aliquots were withdrawn, immediately mixed with 10 μL of 2x SDS loading buffer, boiled for 5 min, and stored at -20 °C. The aliquots were analyzed by SDS-PAGE on a 15% gel. The gel was stained with Coomassie blue dye and scanned on an Odyssey CLx Imager (LI-COR) in the 700-nm channel. The densitometric quantification of the intact protein was performed using the Image studio lite software.

BRET Assay. HEK293T cells were seeded in 6-well plate (6.5 x 10^5 cells/well). Next day, the cells were transfected with pEF-RLUC8-L15-KrasG12D or pEF-RLUC8-L15-KrasG12V[45] and pEF-CRafRBD(1-149)-L15-GFP[45] in a 1:2 ratio (50 ng of KRas and 100 ng of CRafRBD-GFP). The cells were incubated for 24 h at 37 °C. The cells were reseeded in a white 96-well plate (50,000 cells/well) and incubated for 4 h at 37 °C and varying concentrations of MTD4-RBDV or RBDV were added into each well. After incubation for 20–24 h, coelenterazine 400a was added to a final concentration of 10 μM and the BRET signal was recorded on a Tecan Infinite M1000 Pro plate reader.

Western Blotting. HEK293T cells were seeded in a 6-well plate (6.0 x 10^5 cells/well). The cells were treated with different concentrations of PTP1B, MTD4-PTP1B, or MTD4-PTP1B(C215S) for 6 h in serum-free media. The cells were stimulated for 10 min with EGF (50

ng/mL) and then treated with 2 mM sodium pervanadate for 5 min. The cells were washed with PBS and lysed by incubation in 100 μL of Pierce RIPA buffer containing 2 mM sodium pervanadate, phosphatase inhibitors, and protease inhibitors for 30 min on ice. The lysate was centrifuged at 16,000g for 20 min and the total protein concentration of each sample was measured using the BCA protein assay kit (Thermo). Equal amounts of total protein samples were loaded onto each lane of a 10% SDS-PAGE gel. After separation by electrophoresis (120 V, 2.5 h), the proteins were electrophoretically transferred onto a 0.45-μm nitrocellulose membrane using a Bio-Rad trans-blot turbo transfer system (2.5 A and 25 V for 20 min). The membrane was blocked with 5% BSA in TBST (20 mM Tris, pH 7.5, 150 mM NaCl, 0.1% (v/v) Tween-20) at RT for 1 h. The membrane was incubated with anti-pY antibody 4G10 (1:1,000 dilution, MilliporeSigma, #05-321) at 4 °C overnight. The membrane was washed with TBST three times and incubated with fluorescently labeled anti-mouse secondary antibodies (1:10,000 dilution, LI-COR, #925-32210) at room temperature for 2 h. The membrane was washed three times with TBST and fluorescence signals were recorded. The signals were normalized to the total protein per lane of the cell lysate.

MiaPaCa-2 cells were seeded in 12-well plate (1.5 x 10^5 cells/well). The following day, the cells were treated with varying concentrations of MTD4-RBDV (or control) for 4 h in DMEM supplemented with 10% FBS and 1% penicillin/streptomycin. The cells were stimulated with EGF (50 ng/mL) for 10 min and the cell lysates were analyzed by gel electrophoresis on a 12% SDS-PAGE gel. The gel was transferred onto a 0.45 μm nitrocellulose membrane at 90 V, 4 °C for 2 h. The membrane was blocked as described and incubated with anti-MEK (Cell Signaling Technology (CST, #9122), anti-pMEK (CST, #9121), anti-Akt (CST, #9272), anti-pAkt(T308) (CST, #9275), anti-p-AKT(S473) and/or anti-GAPDH monoclonal antibodies at a 1:1000 dilution overnight at 4 °C. The membranes were washed three times with TBST and incubated with fluorescently labeled secondary antibodies (LI-COR) at room temperature for 2 h. The membrane was washed with TBST three times and the fluorescent signals were recorded.

GM01661 and GM00525 cells were seeded in a 12-well plate (1.5 x 10^5 cells/well). The cells were grown for 72 h and treated with ASL-MTD4, ASL, or vehicle for 6 h in DMEM supplemented with 1% FBS. The cells were exhaustively washed with DPBS and incubated for 48 h in DMEM supplemented with 10% FBS. The cells were trypsinized, harvested, lysed, run on SDS-PAGE, transferred onto the membrane, and blotted against human specific anti-ASL antibody (Abcam, ab201025). The protein was detected using fluorescently labeled anti-rabbit antibody. All nitrocellulose membranes were scanned in the 800 nm channel on a LICOR Odyssey CLx or Chemidoc MP imaging system (BioRad).

Annexin V/PI Staining. H358 cells were seeded into a 12-well plate (1.0 × 10^5 cells/well). The following day, the cells were washed with DPBS and treated with varying concentrations of MTD4-RBDV in complete growth medium (10% FBS) for 24 h. The media from the treated wells was collected. Subsequently, the adherent cells were thoroughly washed with DPBS and harvested using trypsin. To ensure the recovery of all cells (some of which became detached during the experiment), the treatment media, the DPBS wash, and the harvested cells derived from the same sample well were combined and centrifuged at 300g and 4 °C for 5 min. The cells were washed twice with DPBS to remove any remaining trypsin. Next, Annexin V staining was performed following manufacturer's protocol (Invitrogen). Briefly, the cell pellet was resuspended in 100 μL of 1× annexin-binding buffer and incubated at room temperature for 15 min with 5 μL of

AlexaFluor® 488-labeled Annexin V and 1 µL of propidium iodide (PI, 100 µg/mL). Finally, 400 µL of 1× annexin-binding buffer was added to each tube immediately before analysis on a BD LSR Fortessa flow cytometer for fluorescence emission at 530 and 575 nm.

Cell Viability Assay. H358, SW480, MiaPaCa-2, H1915, MDA-MB-468, HCT116, or H1299 cells were seeded (5000 cells/well) in opaque 96-well plates. The following day, the cells were treated with serially diluted MTD4-RBDV solutions (or PBS and control proteins) in complete growth medium (10% FBS) for 72 h. Cell Titer Glo reagent (Promega) was added, and the luminescence signal was measured on a Tecan Infinite M1000 Pro plate reader by following the manufacturer's protocol. All viability values reported are relative to that of PBS-treated cells.

Argininosuccinate Assay. GM01661 and GM00525 cells were seeded in 6-well plates at a density of 3.0×10^5 cells per well and cultured for 72 h in DMEM supplemented with 10% FBS. Cells were then treated with ASL, ASL-MTD4, or vehicle control for 6 h in DMEM containing 1% FBS, followed by three washes with DPBS. Cells were subsequently incubated for 48 h in DMEM with 10% FBS. After incubation, cells were harvested using trypsin, washed with DPBS, and lysed in RIPA buffer. Proteins in the lysate were precipitated using formic acid and acetonitrile. The mixture was centrifuged, and the supernatant was collected for LC-MS/MS analysis using a Thermo Orbitrap Exploris 240 mass spectrometer. Separation of urea cycle metabolites was performed using a Waters ACQUITY UPLC BEH Amide column, with a mobile phase of 10 mM ammonium formate in acetonitrile:water (95:5, v/v) containing 0.4% acetic acid. Argininosuccinate quantification was performed by LC-MS/MS using known amounts of argininosuccinate (400-4000 ng/mL) to generate a linear response curve. Concentrations in samples were calculated from the integrated peak areas using this calibration curve.

Animal Studies. All animal experiments were performed in compliance with the institutional animal care guidelines and according to committee-approved protocols. Ai14 reporter mice (The Jackson Laboratory; B6.Cg-Gt(ROSA)26Sor$^{tm14(CAG-tdTomato)Hze/J}$, stock no. 007914) carry a loxP-flanked STOP cassette preventing transcription of a CAG promoter-driven tdTomato reporter gene. Ai14 mice were intravenously injected with equimolar amounts of endotoxin-free MEC or EC (100 µL of 98 µM solution) or vehicle (buffer only) and euthanized 72 h post-injection. Tissues from the brain, heart, lung, kidney, liver, quadricep muscle, and injection site were harvested, and half of the organ was embedded in optimal cutting temperature (OCT) compound. The OCT embedded blocks for kidney and injection site were sent to iHisto Inc. for slicing and slide preparation. The processed slides were imaged on Olympus VS200 research slide scanner under a 20x objective to measure the Tdtomato fluorescence in the tissues. QuPath and ImageJ were used for image processing and analysis. Fresh tissues collected from the mice were enzymatically dissociated at 37 °C (300 rpm for 30 min) using DPBS supplemented with a mixture of collagenase types I, II, and IV, collagenase D (0.5 mg/mL), Dispase II (1 mg/mL), and DNase I (50 µg/mL). The digested tissue was passed through a 70 µm cell strainer to obtain a single-cell suspension. Cells from all organs were treated with RBC lysis buffer on ice for 5 min, washed with DPBS, and analyzed by flow cytometry.

C57BL/6J mice were injected with a single dose of WT-ASL or ASL-MTD4 or buffer via tail vein. Serum was collected at different time-points to study the pharmacokinetics of the proteins. Four hours post-injection, liver, kidney, lung, heart, quadricep muscle, gastrocnemius muscle,

brain, spleen, bone marrow, and injection site were collected. The tissues were lysed to perform immunoblotting against ASL as described before. Part of liver, kidney, heart, lung, and spleen were embedded into OCT compound. The tissues were sliced, washed with DPBS to remove OCT compound, treated with TrueView autofluorescence quenching kit (Vector, SP-8400), washed with DPBS, and incubated with anti-human ASL antibody (Invitrogen, PA5-117795) for 1 h at 1:100 dilution. The sections were washed and incubated with goat anti-rabbit IgG H&L secondary antibody labeled with Alexa Fluor® 488 for 1 h at 1:500 dilution. Finally, the sections were exhaustively washed, counterstained with DAPI, and imaged using MICA fluorescence scanner.

Ex Vivo Gene Editing. Primary lung cells from Ai14 mice were seeded in 6-well plates at a density of 1×10^5 cells/well. The following day, the cells were washed with DPBS and treated with varying concentrations of MEC, EC, or vehicle (buffer only) for 24 h in serum-free media. The cells were washed twice with DPBS and incubated in complete growth media for 48 h. The cells were then washed twice with cold DPBS and analyzed on a BD FACS LSR II flow cytometer. The percentage of tdTomato$^+$ cells (λ_{ex} = 581 nm) was plotted against the protein concentration.

References

57. Liu, H.; Naismith, J. H. An Efficient One-Step Site-Directed Deletion, Insertion, Single, and Multiple-Site Plasmid Mutagenesis Protocol. *BMC Biotechnol.* **2008**, *8*, 91.

58. Bolte, S.; Cordelières, F. P. A Guided Tour into Subcellular Colocalization Analysis in Light *Microscopy. J. Microsc.* **2006**, 224 (3), 213–232.

59. Lavinder, J. J.; Hari, S. B.; Sullivan, B. J.; Magliery, T. J. High-Throughput Thermal Scanning: A General, Rapid Dye-Binding Thermal Shift Screen for Protein Engineering. *J. Am. Chem. Soc.* **2009**, *131*, 3794–3795.

60. Sankaranarayanan, S.; De Angelis, D.; Rothman, J. E.; Ryan, T. A. The use of pHluorins for optical measurements of presynaptic activity. *Biophys. J.* **2000***,* *79*, 2199-2208.

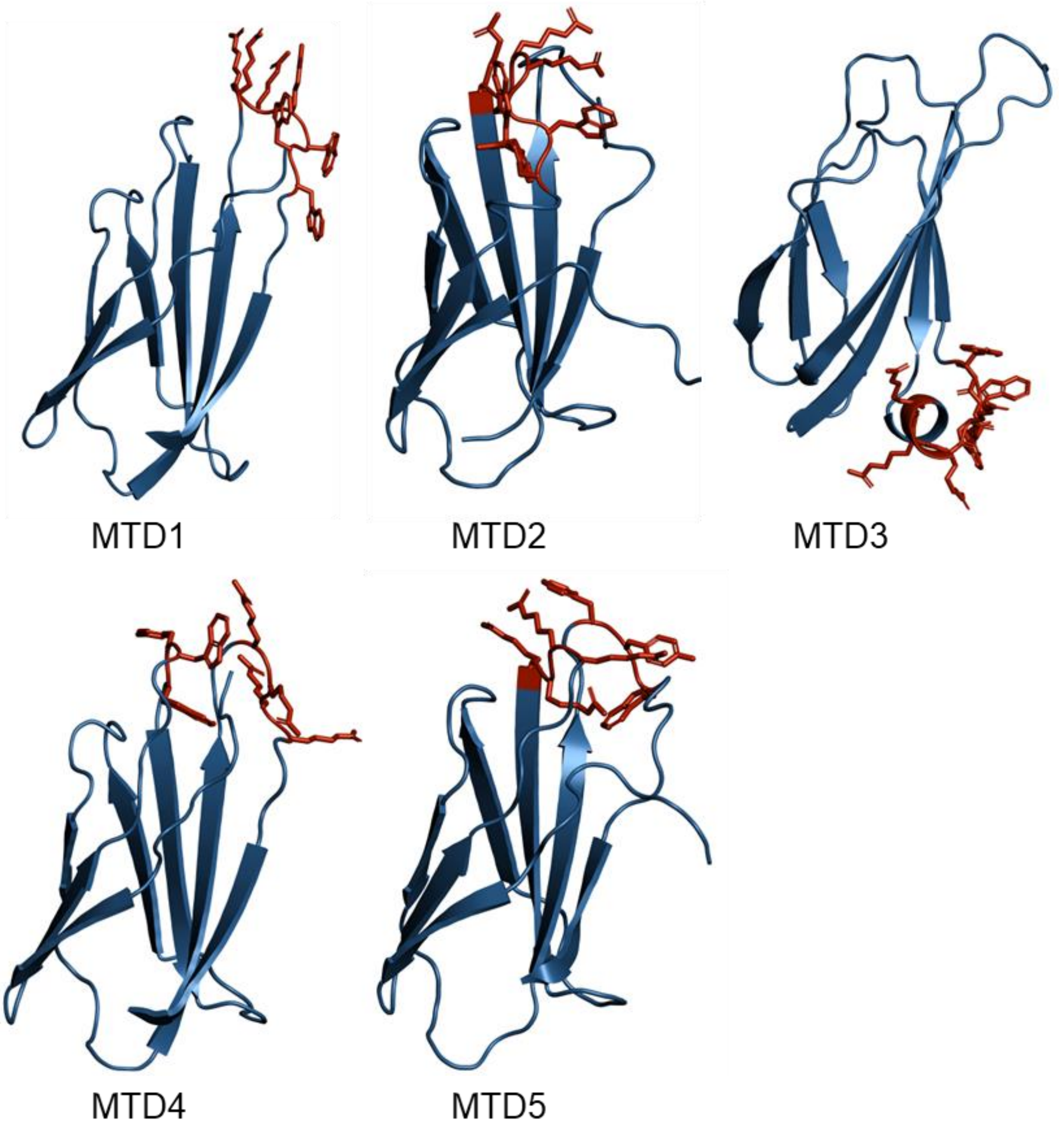

Figure S1. Predicted 3D structures of MTD1–5 by Phyre2 (an online structure prediction software based on sequence homology), with the CPP residues (and their side chains) highlighted in red. Structural analysis was performed on PDB structures with highest confidence obtained from Phyre2 using PyMOL™ 2.5.5 (Molecular Graphics System, Schrödinger, LLC).

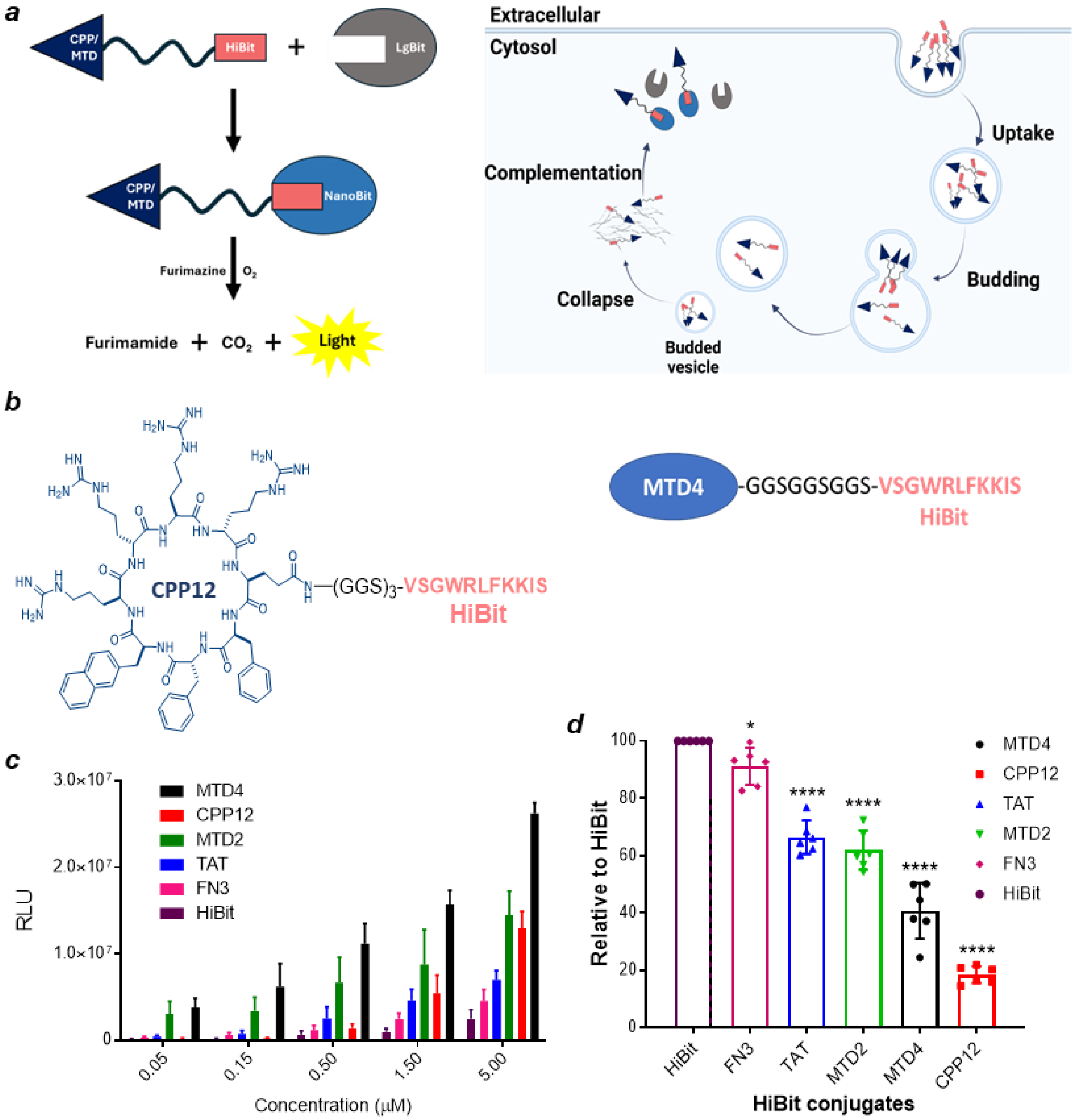

Figure S2. NanoLuc complementation assay and the relative complementation efficiencies of different HiBit conjugates. ***(a)*** Schematic representation of the NanoLuc complementation assay. LgBit is expressed inside the cytosol of HEK293T cells. Upon cytosolic entry (presumably by endocytosis and endosomal escape via VBC), HiBit binds to LgBit and forms a functional luciferase inside the cell. (***b***) Structures of MTD4-HiBit and CPP12-HiBit. **(*c*)** Raw cytosolic delivery efficiencies of MTDs and controls as measured by the NanoLuc complementation assay. Relative luminescence units (RLU) in HEK293T cells are plotted as a function of peptide/protein concentration (n = 6). **(*d*)** Complementation efficiencies of FN3-, Tat-, MTD2-, MTD4-, and CPP12-HiBit conjugates (relative to HiBit, 100%). The data shown represents the mean ± SD of six independent experiments (n = 6). One-tailed paired t-test was performed between HiBit and all HiBit conjugates to obtain the *p*-values, * $p \leq 0.05$, and **** $p \leq 0.0001$.

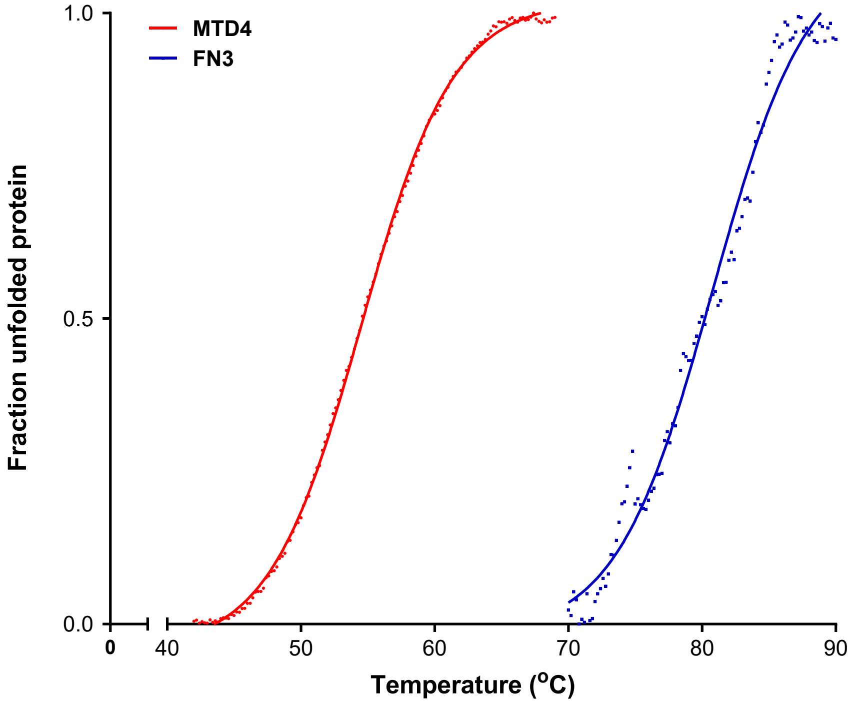

Figure S3. Thermal denaturation profiles of FN3 and MTD4 as monitored by HTTS. The melting temperature (T_m) of a protein is defined as the temperature at which half of the protein is in the unfolded state. The T_m values reported represent the mean ± SD of three independent experiments (n = 3).

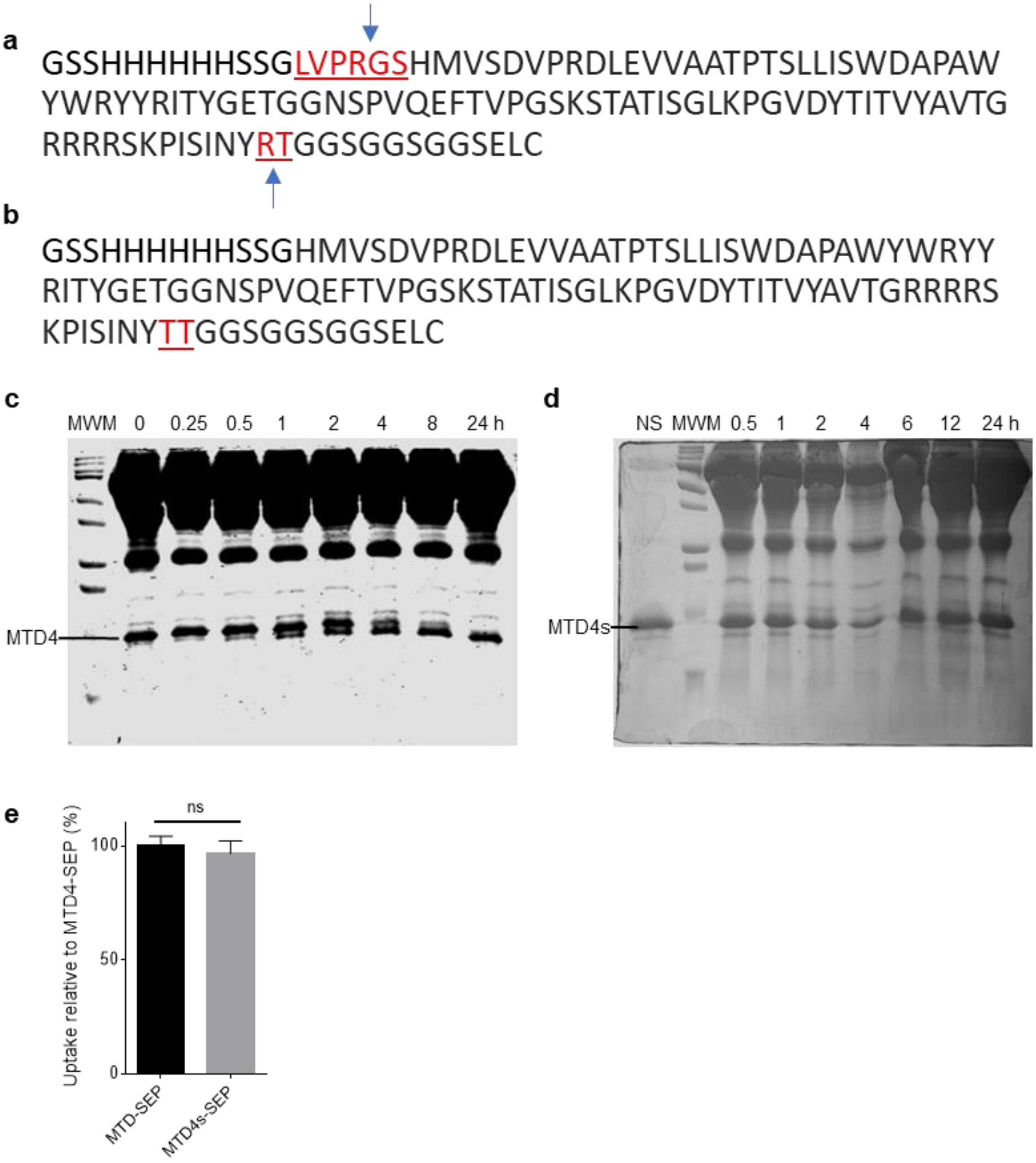

Figure S4. Serum stability of MTD4 and MTD4s. (**a)** Amino acid sequence of MTD4, with the proteolytic cleavage sites indicated by arrows. (**b**) Amino acid sequence of MTD4s. (**c**) Coomassie blue-stained SDS-PAGE gel showing the time-dependent proteolysis of MTD4 (13.2 kD) into a ~12 kD species ($t_{1/2}$ ~3 h). (**d**) SDS-PAGE gel showing MTD4s (12.6 kDa) after varying times of incubation in human serum. No significant cleavage was observed after 24 h of incubation ($t_{1/2}$ >24 h). MWM, molecular weight markers; NS, no serum (MTD4s only). (**e**) Comparison of the cytosolic entry efficiencies of MTD4-SEP and MTD4s-SEP in HeLa cells following a 2-h incubation with 250 nM protein, as measured by flow cytometry. SEP (super ecliptic pHluorin) is a pH-sensitive variant of green fluorescent protein (pKa ~7.2), which is fluorescent in the cytosol but nonfluorescent inside the acidic endosome or lysosome.[60] Data represent the mean ± SD of three independent experiments (n = 3). ns, not significant, as determined by a paired Student's *t*-test.

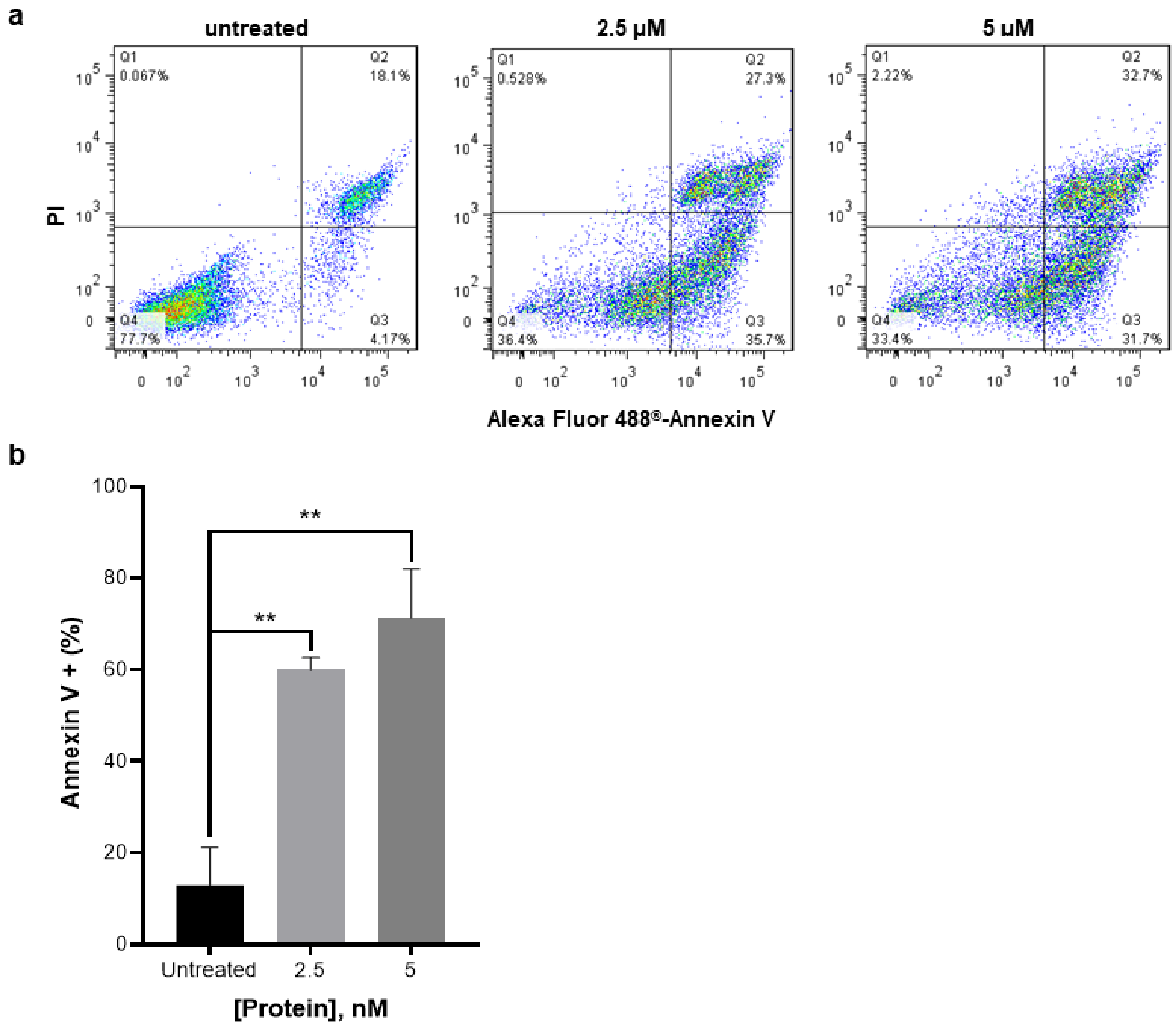

Figure S5. Flow cytometry analysis of H358 cells treated with MTD4-RBDV. **(a)** H358 cells were treated with 0 (buffer only), 2.5, or 5 µM MTD4-RBDV for 24 h in DMEM supplemented with 10% FBS. Prior to flow cytometry analysis, the cells were stained with AlexaFluor® 488-Annexin V and propidium iodide. The analysis was performed for at least 10,000 cells. **(b)** Percentage of annexin V-positive cells (in Q2 and Q3) as a function of MTD4-RBDV concentration. Data shown represent the mean ± SD from three independent experiments (n = 3).

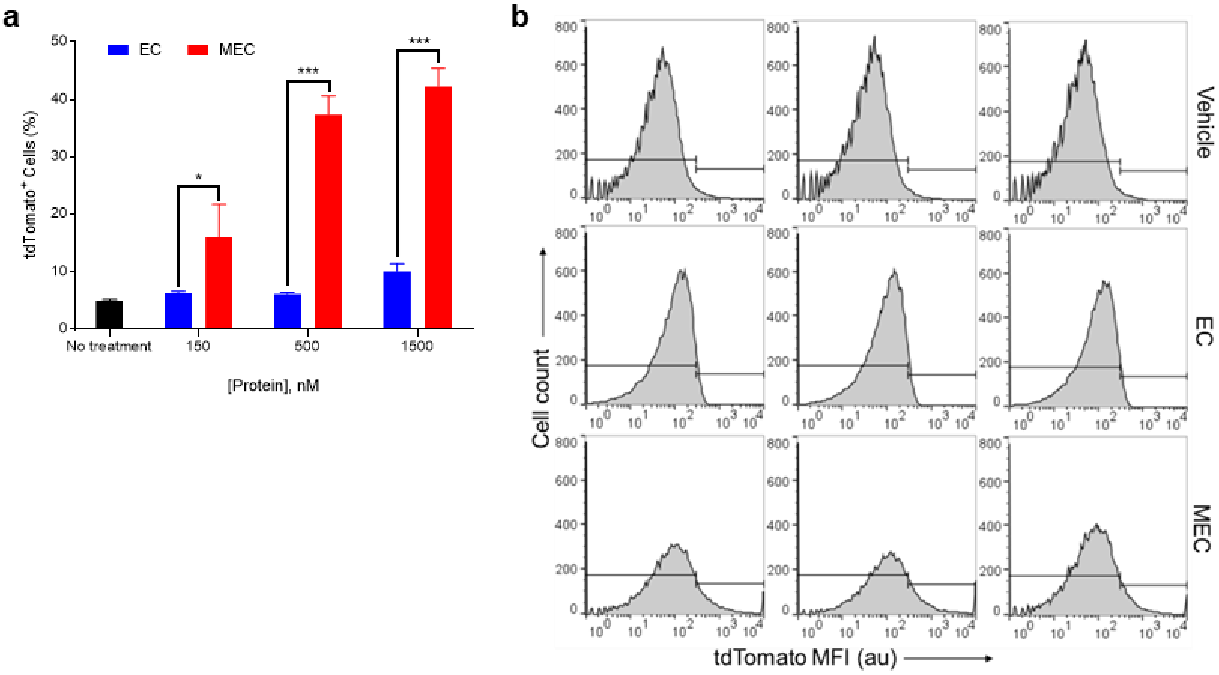

Figure S6. Flow cytometry analysis of tdTomato expression following treatment with vehicle (no treatment), EC, or MEC. **(a)** Percentage of tdTomato-positive cells after *ex vivo* treatment of primary lung cells isolated from Ai14 mice with increasing concentrations of EC or MEC (n = 4). Statistical analysis was performed using a one-tailed paired Student's t-test between EC and MEC groups (** $p \leq 0.01$, *** $p \leq 0.001$, **** $p \leq 0.0001$). **(b)** Histograms showing tdTomato fluorescence intensity (MFI, arbitrary units) in dissociated kidney cells for mice injected with vehicle (top row), EC (middle row), or MEC (bottom row). Each column corresponds to an independent biological replicate. Gates were set such that ≤1.5 % of vehicle-treated cells were above the threshold, and identical gating parameters were applied to all samples (raw data corresponding to Figure 6c).

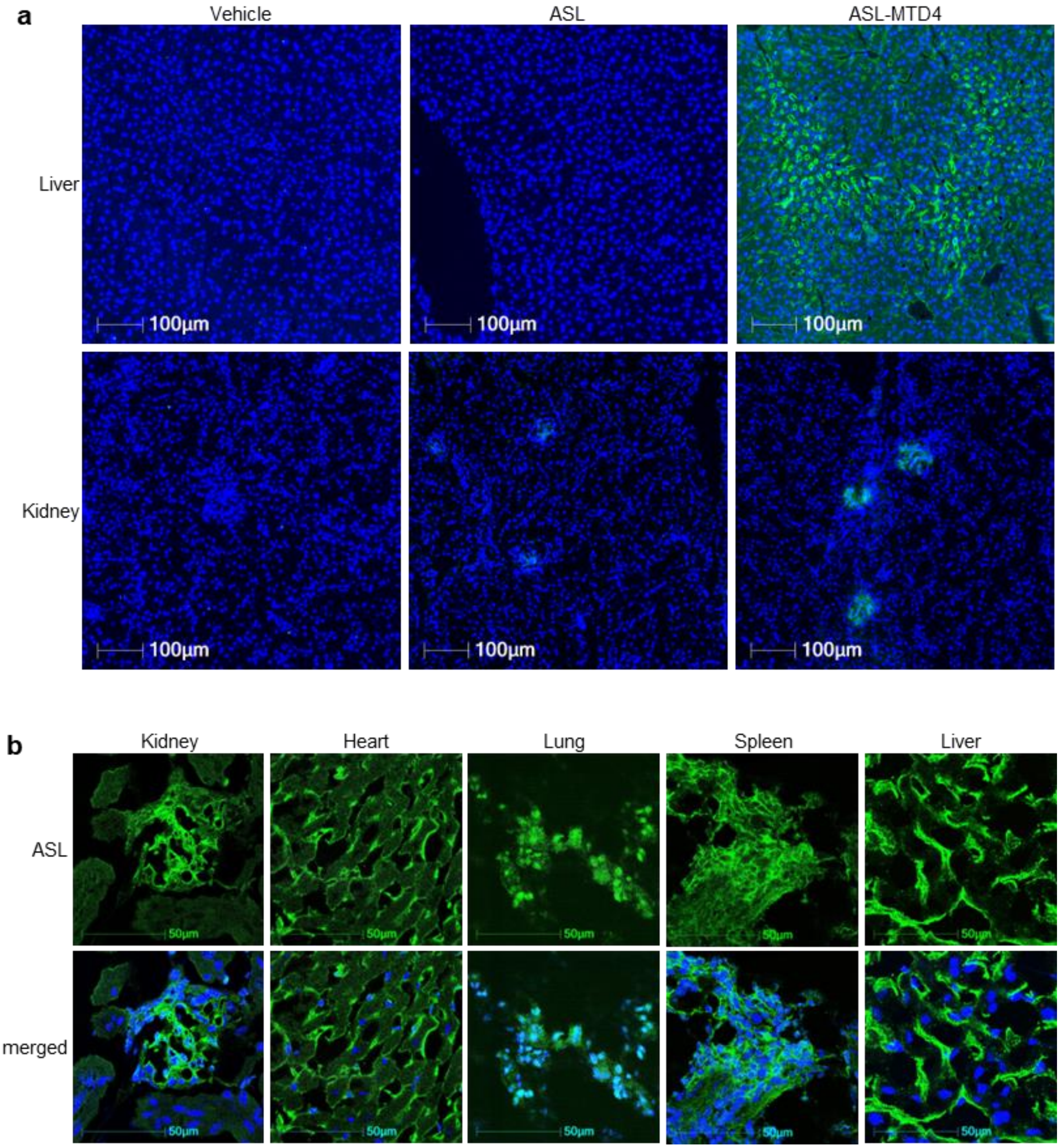

Figure S7. Cellular uptake and intracellular distribution of ASL-MTD4 in different mouse organs. (**a**) Confocal microscopic images liver and kidney cryosections collected 4 h after intravenous injection of vehicle (buffer only) or equimolar amount of ASL or ASL-MTD4 (~30 µM, 100 µL). Tissues were immunostained with anti-human ASL antibody (green) and counterstained with DAPI (blue). Scale bar, 100 µm. (**b**) High-magnification confocal images of mice organ sections from Fig. 7c., Scale bar, 50 µm. Images are representative of n = 3 biological replicates.

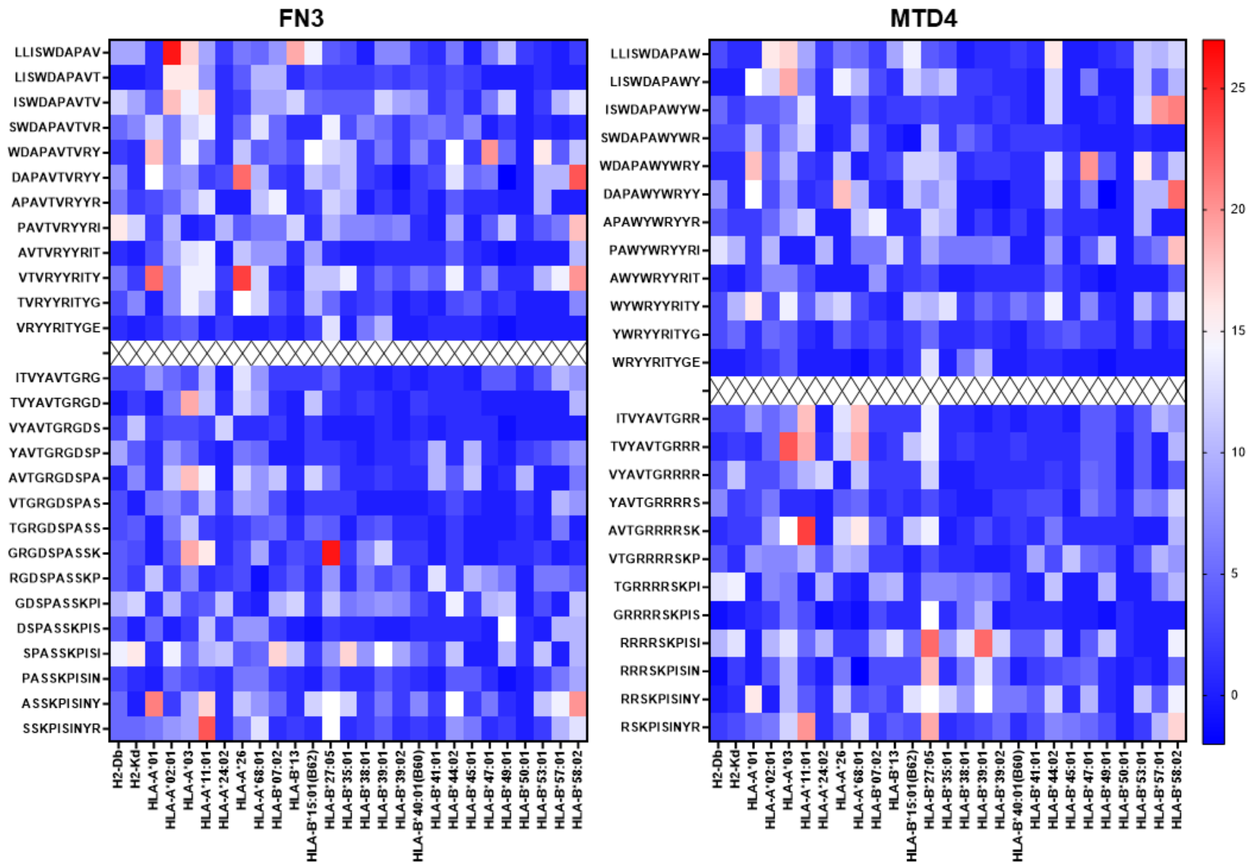

Figure S8. Heatmap showing predicted T cell immunogenicity scores of epitopes in FN3 and MTD4. In silico analysis was performed for all possible octapeptides, nonapeptides, decapeptides, undecapeptides, and pentadecapeptides bound to human major histocompatibility complexes. Overall, both FN3 and MTD4 have low immunogenicity scores and the mutations in the BC and FG loops of MTD4 do not significantly increase the immunogenicity score. Data shown represent the scores of decapeptides derived from the BC and FG loop regions of FN3 and MTD4.

Figure S9. Structures and analytical data of peptides used in this work.

HiBit

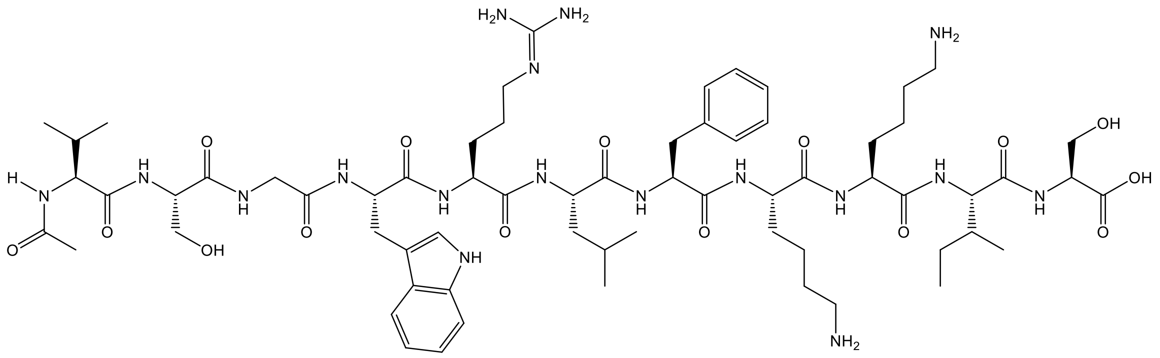

Purity assessment by UPLC (214 nm):

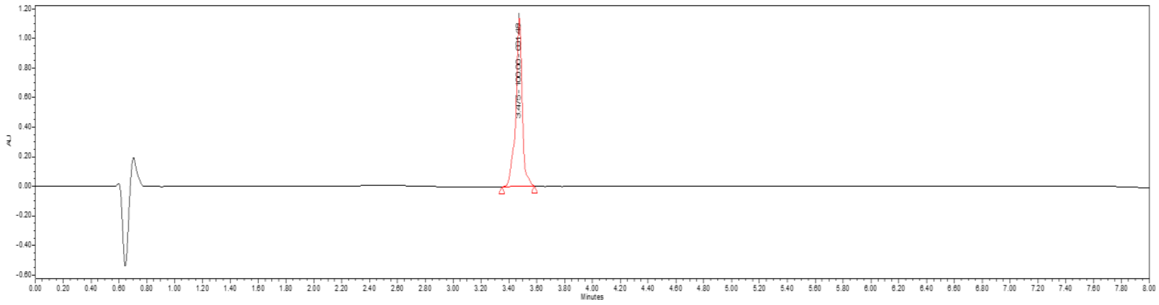

ESI-MS:

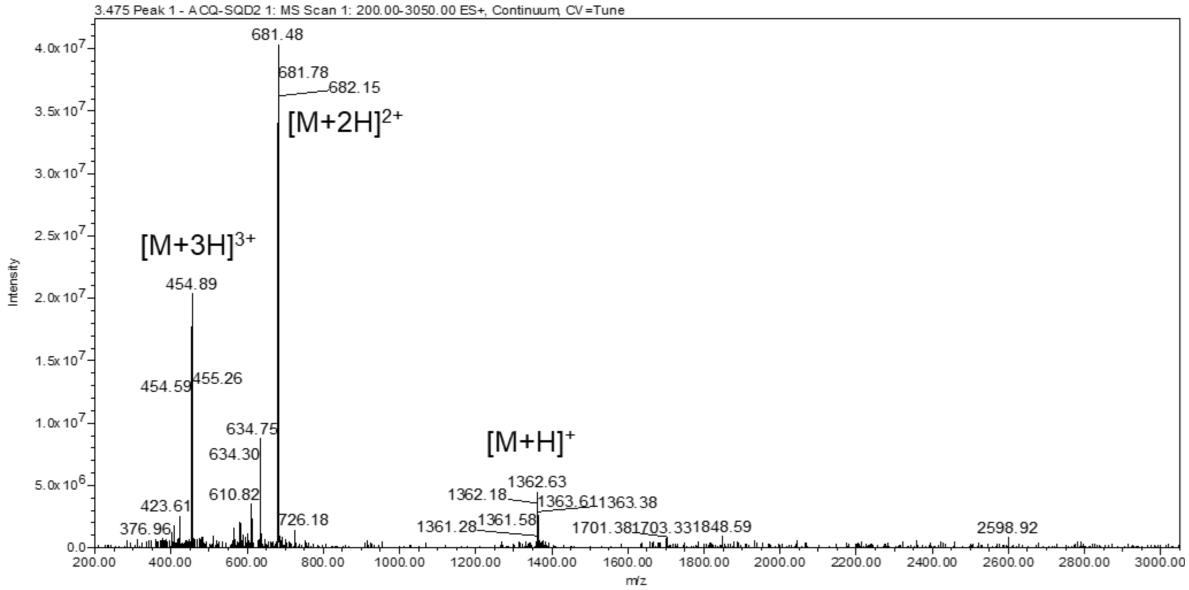

Calculated $[M+3H]^{3+}$: 454.93; observed 454.89
Calculated $[M+2H]^{2+}$: 681.89; observed 681.78
Calculated $[M+H]^{1+}$: 1362.78; observed 1362.63

TAT-HiBit

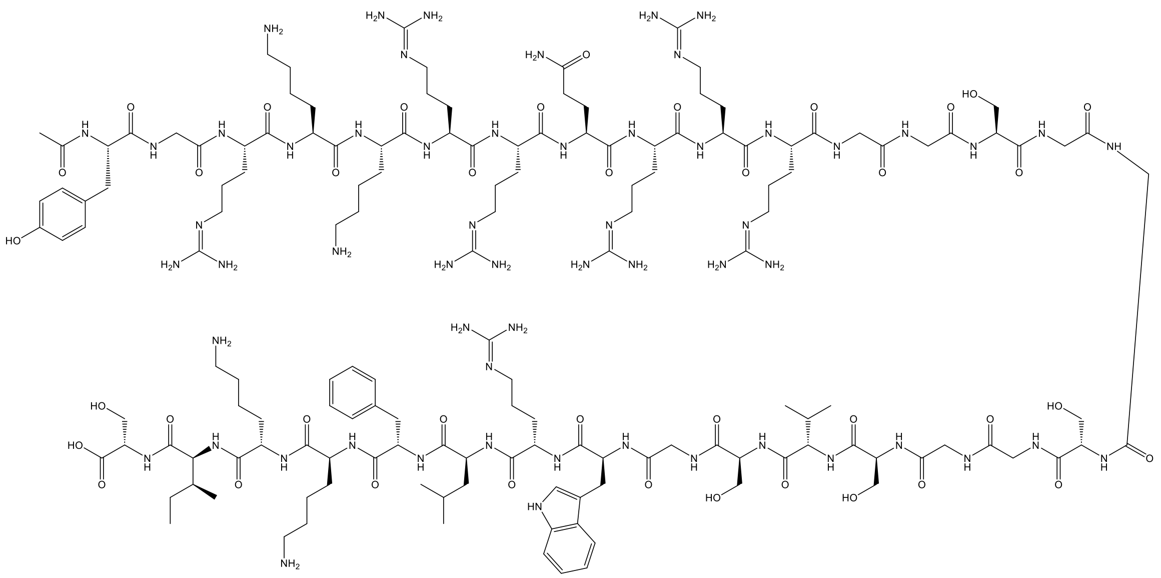

Purity assessment by UPLC (214 nm):

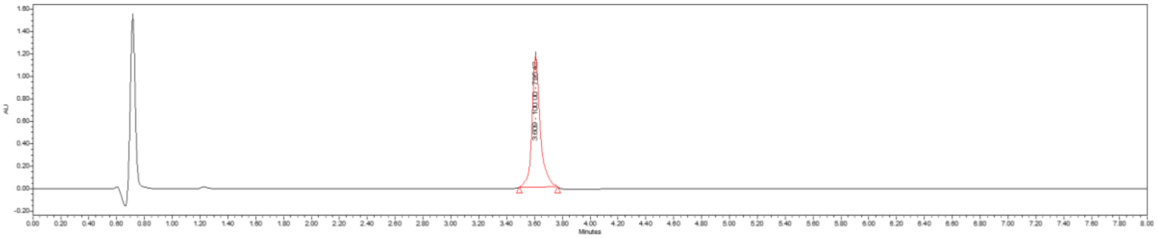

ESI-MS:

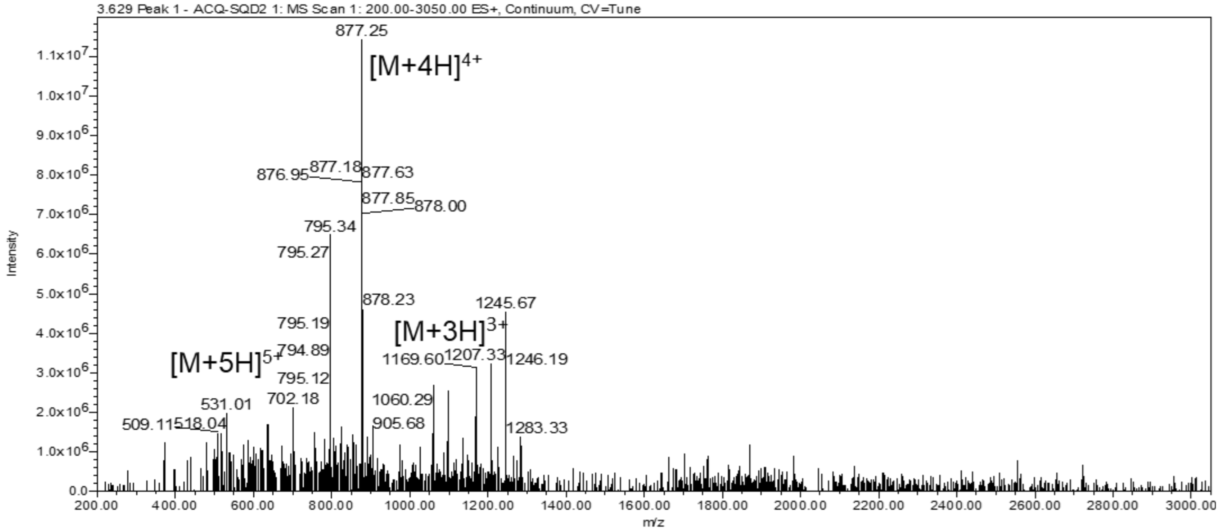

Calculated $[M+5H]^{5+}$: 702.19; observed 702.18

Calculated $[M+4H]^{4+}$: 877.49; observed 877.25

Calculated $[M+3H]^{3+}$: 1169.65; observed 1169.29

CPP12-HiBit

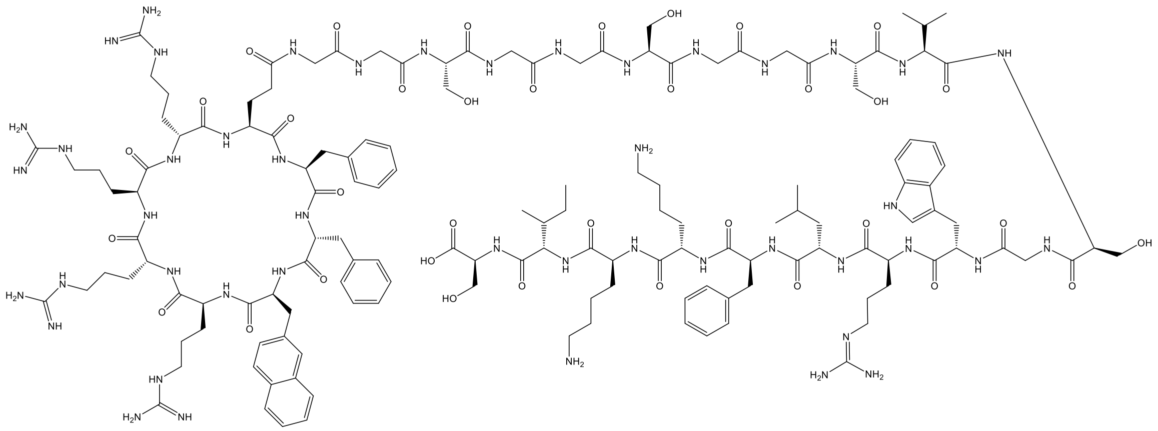

Purity assessment by UPLC (214 nm):

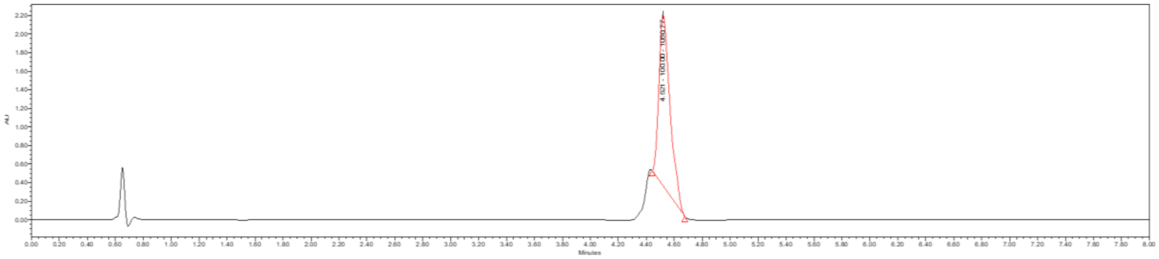

ESI-MS:

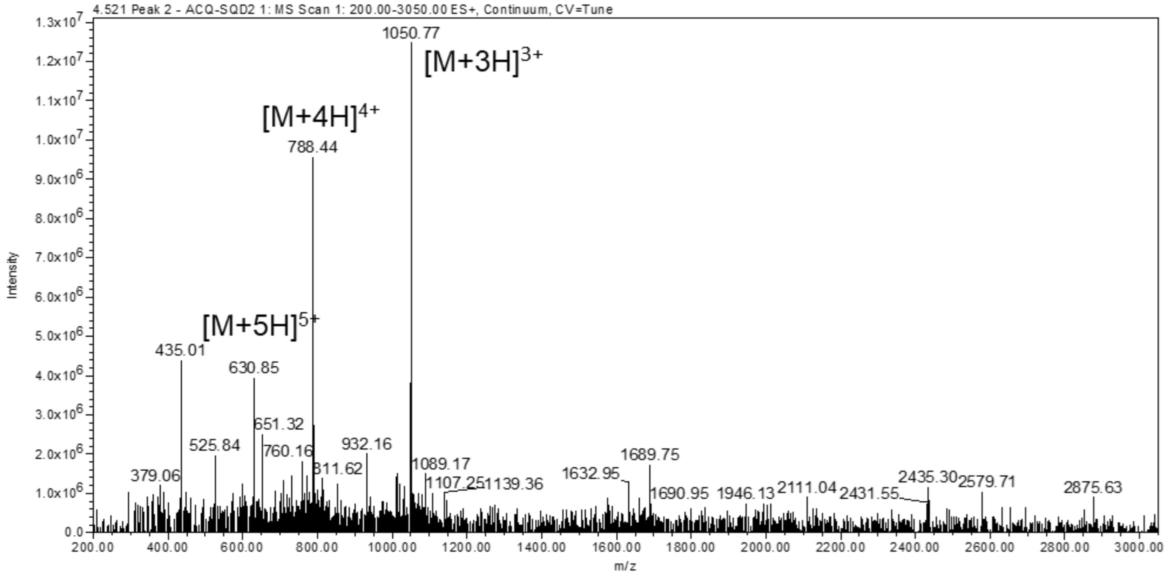

Calculated $[M+5H]^{5+}$: 630.93; observed 630.85
Calculated $[M+4H]^{4+}$: 788.41; observed 788.44
Calculated $[M+3H]^{3+}$: 1050.88; observed 1050.77

Table S1. Sequences of protein and DNA primer used in this work

Protein	*Sequence*
FN3	MGSSHHHHHHSSGLVPRGSHMVSDVPRDLEVVAATPTSLLISWDAPAVT<br>VRYYRITYGETGGNSPVQEFTVPGSKSTATISGLKPGVDYTITVYAVTGR<br>GDSPASSKPISINYRTGGSGGSGGSELC
MTD1	MGSSHHHHHHSSGLVPRGSHMVSDVPRDLEVVAATPTSLLISWDAPAVT<br>VRYYRITYGETGGNSPVQEFTVPGSKSTATISGLKPGVDYTITVYAVTGR<br>RRRWWWSKPISINYRTGGSGGSGGSELC
MTD2	MGSSHHHHHHSSGLVPRGSHMVSDVPRDLEVVAATPTSLLISWDAPWW<br>WRRRRYYRITYGETGGNSPVQEFTVPGSKSTATISGLKPGVDYTITVYAV<br>TGRGDSPASSKPISINYRTGGSGGSGGSELC
MTD3	MGSSHHHHHHSSGLVPRGSHMVSDVPRDLEVVAATPTSLLISWDAPAVT<br>VRYYRITYGETGGRRRRWWWVQEFTVPGSKSTATISGLKPGVDYTITVY<br>AVTGRGDSPASSKPISINYRTGGSGGSGGSELC
MTD4	MGSSHHHHHHSSGLVPRGSHMVSDVPRDLEVVAATPTSLLISWDAPAW<br>YWRYYRITYGETGGNSPVQEFTVPGSKSTATISGLKPGVDYTITVYAVTG<br>RRRRSKPISINYRTGGSGGSGGSELC
MTD4s	MGSSHHHHHHSSGHMVSDVPRDLEVVAATPTSLLISWDAPAWYWRYYR<br>ITYGETGGNSPVQEFTVPGSKSTATISGLKPGVDYTITVYAVTGRRRRSKP<br>ISINYTTGGSGGSGGSELC
MTD5	MGSSHHHHHHSSGLVPRGSHMVSDVPRDLEVVAATPTSLLISWDAPARR<br>RRYYRITYGETGGNSPVQEFTVPGSKSTATISGLKPGVDYTITVYAVTGW<br>YWRSKPISINYRTGGSGGSGGSELC
FN3-HiBit	MGSSHHHHHHSSGLVPRGSHMVSDVPRDLEVVAATPTSLLISWDAPAVT<br>VRYYRITYGETGGNSPVQEFTVPGSKSTATISGLKPGVDYTITVYAVTGR<br>GDSPASSKPISINYRTGGSGGSGGSVSGWRLFKKIS
MTD2-HiBit	MGSSHHHHHHSSGLVPRGSHMVSDVPRDLEVVAATPTSLLISWDAPWW<br>WRRRRYYRITYGETGGNSPVQEFTVPGSKSTATISGLKPGVDYTITVYAV<br>TGRGDSPASSKPISINYRTGGSGGSGGSVSGWRLFKKIS
MTD4-HiBit	MGSSHHHHHHSSGLVPRGSHMVSDVPRDLEVVAATPTSLLISWDAPAW<br>YWRYYRITYGETGGNSPVQEFTVPGSKSTATISGLKPGVDYTITVYAVTG<br>RRRRSKPISINYRTGGSGGSGGSVSGWRLFKKIS
PTP1B	MEMEKEFEQIDKSGSWAAIYQDIRHEASDFPCRVAKLPKNKNRNRYRDV<br>SPFDHSRIKLHQEDNDYINASLIKMEEAQRSYILTQGPLPNTCGHFWEMV<br>WEQKSRGVVMLNRVMEKGSLKCAQYWPQKEEKEMIFEDTNLKLTLISE<br>DIKSYYTVRQLELENLTTQETREILHFHYTTWPDFGVPESPASFLNFLFKV<br>RESGSLSPEHGPVVVHCSAGIGRSGTFCLADTCLLLMDKRKDPSSVDIKK

	VLLEMRKFRMGLIQTADQLRFSYLAVIEGAKFIMGDSSVQDQWKELSHE DLEPPPEHIPPPPRPPKRILEPHNVDKLAAALEHHHHHH
MTD4-PTP1B	MGSSHHHHHHSSGLVPRGSHMVSDVPRDLEVVAATPTSLLISWDAPAW YWRYYRITYGETGGNSPVQEFTVPGSKSTATISGLKPGVDYTITVYAVTG RRRRSKPISINYRTEAAAKEAAAKELMEMEKEFEQIDKSGSWAAIYQDIR HEASDFPCRVAKLPKNKNRNRYRDVSPFDHSRIKLHQEDNDYINASLIKM EEAQRSYILTQGPLPNTCGHFWEMVWEQKSRGVVMLNRVMEKGSLKCA QYWPQKEEKEMIFEDTNLKLTLISEDIKSYYTVRQLELENLTTQETREILH FHYTTWPDFGVPESPASFLNFLFKVRESGSLSPEHGPVVVHCSAGIGRSGT FCLADTCLLLMDKRKDPSSVDIKKVLLEMRKFRMGLIQTADQLRFSYLA VIEGAKFIMGDSSVQDQWKELSHEDLEPPPEHIPPPPRPPKRILEPHN
MTD4-PTP1B (C215S)	MGSSHHHHHHSSGLVPRGSHMVSDVPRDLEVVAATPTSLLISWDAPAW YWRYYRITYGETGGNSPVQEFTVPGSKSTATISGLKPGVDYTITVYAVTG RRRRSKPISINYRTEAAAKEAAAKELMEMEKEFEQIDKSGSWAAIYQDIR HEASDFPCRVAKLPKNKNRNRYRDVSPFDHSRIKLHQEDNDYINASLIKM EEAQRSYILTQGPLPNTCGHFWEMVWEQKSRGVVMLNRVMEKGSLKCA QYWPQKEEKEMIFEDTNLKLTLISEDIKSYYTVRQLELENLTTQETREILH FHYTTWPDFGVPESPASFLNFLFKVRESGSLSPEHGPVVVHSSAGIGRSGT FCLADTCLLLMDKRKDPSSVDIKKVLLEMRKFRMGLIQTADQLRFSYLA VIEGAKFIMGDSSVQDQWKELSHEDLEPPPEHIPPPPRPPKRILEPHN
RBDV	MPSKTSNTIRVLLPNQEWTVVKVRNGMSLHDSLMKALKRHGLQPESSAV FRLLHEHKGKKARLDWNTDAASLIGEELQVDFLDHVPLTTHNFARKTFL KLGIHRDGSSGSSLEHHHHHH
MTD4-RBDV	MGSSHHHHHHSSGLVPRGSHMVSDVPRDLEVVAATPTSLLISWDAPAW YWRYYRITYGETGGNSPVQEFTVPGSKSTATISGLKPGVDYTITVYAVTG RRRRSKPISINYRTGGSGGSGGSELKTSNTIRVLLPNQEWTVVKVRNGMS LHDSLMKALKRHGLQPESSAVFRLLHEHKGKKARLDWNTDAASLIGEEL QVDFLDHVPLTTHNFARKTFLKLGIHRD
MEC	MGSSHHHHHHSSGLVPRGSHMVSDVPRDLEVVAATPTSLLISWDAPAW YWRYYRITYGETGGNSPVQEFTVPGSKSTATISGLKPGVDYTITVYAVTG RRRRSKPISINYRTGGSGGSGGSELMVSKGEELFTGVVPILVELDGDVNG HKFSVSGEGEGDATYGKLTLKFICTTGKLPVPWPTLVTTLTYGVQCFSRY PDHMKQHDFFKSAMPEGYVQERTIFFKDDGNYKTRAEVKFEGDTLVNRI ELKGIDFKEDGNILGHKLEYNYNSHNVYIMADKQKNGIKVNFKIRHNIED GSVQLADHYQQNTPIGDGPVLLPDNHYLSTQSALSKDPNEKRDHMVLLE FVTAAGITLGMDELYKSGEQKLISEEDLGGPKKKRKVSNLLTVHQNLPAL PVDATSDEVRKNLMDMFRDRQAFSEHTWKMLLSVCRSWAAWCKLNNR KWFPAEPEDVRDYLLYLQARGLAVKTIQQHLGQLNMLHRRSGLPRPSDS NAVSLVMRRIRKENVDAGERAKQALAFERTDFDQVRSLMENSDRCQDIR NLAFLGIAYNTLLRIAEIARIRVKDISRTDGGRMLIHIGRTKTLVSTAGVEK ALSLGVTKLVERWISVSGVADDPNNYLFCRVRKNGVAAPSATSQLSTRA

	LEGIFEATHRLIYGAKDDSGQRYLAWSGHSARVGAARDMARAGVSIPEI<br>MQAGGWTNVNIVMNYIRNLDSETGAMVRLLEDGDPKKKRKVPKKKRK<br>V
EC	MGSSHHHHHHSSGLVPRGSHMVSKGEELFTGVVPILVELDGDVNGHKFS<br>VSGEGEGDATYGKLTLKFICTTGKLPVPWPTLVTTLTYGVQCFSRYPDH<br>MKQHDFFKSAMPEGYVQERTIFFKDDGNYKTRAEVKFEGDTLVNRIELK<br>GIDFKEDGNILGHKLEYNYNSHNVYIMADKQKNGIKVNFKIRHNIEDGSV<br>QLADHYQQNTPIGDGPVLLPDNHYLSTQSALSKDPNEKRDHMVLLEFVT<br>AAGITLGMDELYKSGEQKLISEEDLGGPKKKRKVSNLLTVHQNLPALPV<br>DATSDEVRKNLMDMFRDRQAFSEHTWKMLLSVCRSWAAWCKLNNRK<br>WFPAEPEDVRDYLLYLQARGLAVKTIQQHLGQLNMLHRRSGLPRPSDSN<br>AVSLVMRRIRKENVDAGERAKQALAFERTDFDQVRSLMENSDRCQDIRN<br>LAFLGIAYNTLLRIAEIARIRVKDISRTDGGRMLIHIGRTKTLVSTAGVEKA<br>LSLGVTKLVERWISVSGVADDPNNYLFCRVRKNGVAAPSATSQLSTRAL<br>EGIFEATHRLIYGAKDDSGQRYLAWSGHSARVGAARDMARAGVSIPEIM<br>QAGGWTNVNIVMNYIRNLDSETGAMVRLLEDGDPKKKRKVPKKKRKV
LgBit-mCherry	MGSSHHHHHHSSGLVPRGSHMVFTLEDFVGDWEQTAAYNLDQVLEQGG<br>VSSLLQNLAVSVTPIQRIVRSGENALKIDIHVIIPYEGLSADQMAQIEEVFK<br>VVYPVDDHHFKVILPYGTLVIDGVTPNMLNYFGRPYEGIAVFDGKKITVT<br>GTLWNGNKIIDERLITPDGSMLFRVTINSGSSGSSGSSMVSKGEEDNMAII<br>KEFMRFKVHMEGSVNGHEFEIEGEGEGRPYEGTQTAKLKVTKGGPLPFA<br>WDILSPQFMYGSKAYVKHPADIPDYLKLSFPEGFKWERVMNFEDGGVVT<br>VTQDSSLQDGEFIYKVKLRGTNFPSDGPVMQKKTMGWEASSERMYPED<br>GALKGEIKQRLKLKDGGHYDAEVKTTYKAKKPVQLPGAYNVNIKLDITS<br>HNEDYTIVEQYERAEGRHSTGGMDELYK

**Primers**	**Sequence (5' to 3')**
MTD1	F- CGTCGACGCTGGTGGTGGAGCAAGCCAATCAGCATTAACTATCGT<br>R- CCACCACCAGCGTCGACGACGGCCGGTCACCGCATA
MTD2	F- TGGTGGTGGCGTCGACGCCGTTATTACCGCATTACGTACGGC<br>R- GCGTCGACGCCACCACCACGGTGCATCCCAGGAAATCAGCAA
MTD3	F- CGTCGACGCCGTTGGTGGTGGGTTCAGGAGTTCACCGTGC<br>R- CCACCACCAACGGCGTCGACGACCACCGGTTTCGCCGTA
MTD4a	F- TGGTATTGGCGTTATTACCGCATTACGTACGGCGAA<br>R- CCAATACCACGCCGGTGCATCCCAGGAAAT
MTD4b	F- CGTCGACGCAGCAAGCCAATCAGCATTAACTATCGT<br>R- GCGTCGACGACGGCCGGTCACCGCATA
MTD5a	F- TGGTATTGGCGTAGCAAGCCAATCAGCATTAACTATCG<br>R- ACGCCAATACCAGCCGGTCACCGCATAGACG
MTD5b	F- CGTCGACGCCGTTATTACCGCATTACGTACGGC<br>R- GCGTCGACGCGCCGGTGCATCCCAG
Thrombin deletion	F- AGCGGCCATATGGTCTCAGATGTACCCAGGGAC<br>R- TGAGACCATATGGCCGCTGCTGTGATGATGATGATGATGG

HiBit insertion	F- GTTAGTGGTTGGCGTCTTTTTAAAAAGATTTCATAAATCGATCGCTCGAGGATCCGGCTG<br>R- TGAAATCTTTTTAAAAAGACGCCAACCACTAACCGAACCGCCAGAGCCACC
PTP1B insertion	F- ATAGCAGAGCTCATGGAGATGGAAAAGGAGTTCGAGCAG<br>R- ACTGGATCCTTAATTGTGTGGCTCCAGGATTCGTTT
(EAAAK)$_2$ insertion primers	F- TCGACGCAGCAAGCCAATCAGCATTAACTATCGTACCGAAGCCGCGGCCAAAGAAGCCGCGGCCAAAGAGCT<br>R- CTTTGGCCGCGGCTTCTTTGGCCGCGGCTTCGGTACGATAGTTAATGCTGATTGGCTTGCTGCG
EGFP-NLS-Cre insertion	F- ATAATAGAGCTCATGGTGAGCAAGGGCGAGGA<br>R- TTGGCACTCGAGTTAATCGCCATCTTCCAGCAGG
EC-Vec insertion	F- ATAATACATATGGTGAGCAAGGGCGAG<br>R- TTGGCACTCGAGTTAATCGCCATCTTCCAGCAG
(NLS)$_2$ insertion	F- CCAAAAAAAAAACGTAAAGTTCCTAAAAAGAAACGTAAGGTATAACTCGAGGATCCGGCTGCTAACAAAGCCCG<br>R- TACCTTACGTTTCTTTTTAGGAACTTTACGTTTTTTTTTTGGATCGCCATCTTCCAGCAGGCGCACCAT
